# Research progress on the role of mitochondria in the process of hepatic ischemia-reperfusion injury

**DOI:** 10.1093/gastro/goae066

**Published:** 2024-06-21

**Authors:** Yujie Zhou, Tao Qiu, Tianyu Wang, Bo Yu, Kang Xia, Jiayu Guo, Yiting Liu, Xiaoxiong Ma, Long Zhang, Jilin Zou, Zhongbao Chen, Jiangqiao Zhou

**Affiliations:** Department of Organ Transplantation, Renmin Hospital of Wuhan University, Wuhan, Hubei, P. R. China; Department of Urology, Renmin Hospital of Wuhan University, Wuhan, Hubei, P. R. China; Department of Organ Transplantation, Renmin Hospital of Wuhan University, Wuhan, Hubei, P. R. China; Department of Urology, Renmin Hospital of Wuhan University, Wuhan, Hubei, P. R. China; Department of Organ Transplantation, Renmin Hospital of Wuhan University, Wuhan, Hubei, P. R. China; Department of Urology, Renmin Hospital of Wuhan University, Wuhan, Hubei, P. R. China; Department of Organ Transplantation, Renmin Hospital of Wuhan University, Wuhan, Hubei, P. R. China; Department of Urology, Renmin Hospital of Wuhan University, Wuhan, Hubei, P. R. China; Department of Organ Transplantation, Renmin Hospital of Wuhan University, Wuhan, Hubei, P. R. China; Department of Urology, Renmin Hospital of Wuhan University, Wuhan, Hubei, P. R. China; Department of Organ Transplantation, Renmin Hospital of Wuhan University, Wuhan, Hubei, P. R. China; Department of Urology, Renmin Hospital of Wuhan University, Wuhan, Hubei, P. R. China; Department of Organ Transplantation, Renmin Hospital of Wuhan University, Wuhan, Hubei, P. R. China; Department of Urology, Renmin Hospital of Wuhan University, Wuhan, Hubei, P. R. China; Department of Organ Transplantation, Renmin Hospital of Wuhan University, Wuhan, Hubei, P. R. China; Department of Organ Transplantation, Renmin Hospital of Wuhan University, Wuhan, Hubei, P. R. China; Department of Organ Transplantation, Renmin Hospital of Wuhan University, Wuhan, Hubei, P. R. China; Department of Organ Transplantation, Renmin Hospital of Wuhan University, Wuhan, Hubei, P. R. China; Department of Organ Transplantation, Renmin Hospital of Wuhan University, Wuhan, Hubei, P. R. China; Department of Urology, Renmin Hospital of Wuhan University, Wuhan, Hubei, P. R. China

**Keywords:** hepatic ischemia-reperfusion injury, mitochondria, MPTP, autophagy, reactive oxygen species, Bcl-2

## Abstract

During liver ischemia-reperfusion injury, existing mechanisms involved oxidative stress, calcium overload, and the activation of inflammatory responses involve mitochondrial injury. Mitochondrial autophagy, a process that maintains the normal physiological activity of mitochondria, promotes cellular metabolism, improves cellular function, and facilitates organelle renewal. Mitochondrial autophagy is involved in oxidative stress and apoptosis, of which the PINK1-Parkin pathway is a major regulatory pathway, and the deletion of PINK1 and Parkin increases mitochondrial damage, reactive oxygen species production, and inflammatory response, playing an important role in mitochondrial quality regulation. In addition, proper mitochondrial permeability translational cycle regulation can help maintain mitochondrial stability and mitigate hepatocyte death during ischemia-reperfusion injury. This mechanism is also closely related to oxidative stress, calcium overload, and the aforementioned autophagy pathway, all of which leads to the augmentation of the mitochondrial membrane permeability transition pore opening and cause apoptosis. Moreover, the release of mitochondrial DNA (mtDNA) due to oxidative stress further aggravates mitochondrial function impairment. Mitochondrial fission and fusion are non-negligible processes required to maintain the dynamic renewal of mitochondria and are essential to the dynamic stability of these organelles. The Bcl-2 protein family also plays an important regulatory role in the mitochondrial apoptosis signaling pathway. A series of complex mechanisms work together to cause hepatic ischemia-reperfusion injury (HIRI). This article reviews the role of mitochondria in HIRI, hoping to provide new therapeutic clues for alleviating HIRI in clinical practice.

## Introduction

The liver receives dual blood supply from the hepatic artery and portal vein. The liver hosts vital biochemical reactions in the human body, primarily powered by mitochondria, constituting about 90%–95% of all energy sources. Mitochondria originated in eukaryotic cells approximately 2 billion years ago and are believed to have formed through endosymbiosis [1, 2]. The widely accepted theory suggests that modern organelles evolved from bacteria and play crucial roles in metabolism, calcium regulation, and cell death. Owing to the liver's critical function and the increasing prevalence of liver injury and failure, liver transplantation has gained popularity in clinical practice [3]. Ischemia-reperfusion injury (IRI) is a clinical condition characterized by cellular damage following organ ischemia and hypoxia, exacerbated upon reoxygenation. During the liver transplantation, IRI occurs inevitably in the process of harvesting, storage, and reperfusion, which is one of the important causes of complications and death in liver transplant patients. Hepatic ischemia-reperfusion injury (HIRI) can be classified as hot or cold according to the temperature at which it occurs. Hot ischemia-reperfusion is most likely to occur in cases of portal embolism, hepatic resection, and shock, whereas the cold ischemia-reperfusion process usually occurs during perfusion and the preservation of the donor liver [4, 5]. The pathogenesis of HIRI is complex and complicated, involving a variety of important physiological activities such as inflammatory responses, oxidative stress, mitochondrial damage, lipid peroxidation, endoplasmic reticulum stress, inflammatory factor release, neutrophil infiltration, calcium overload, cellular autophagy, and apoptosis.

HIRI significantly impairs mitochondrial function, leading to severe damage to the cytoskeleton, membrane structure, and overall cell function, with profound consequences for metabolic and physiological processes. Mitophagy encompasses multiple signaling pathways, with particular focus on the extensively studied PINK1/Parkin pathway and other non-PINK1/Parkin-dependent pathways [6]. Impaired function of the mitochondrial membrane permeability transition pore (MPTP) affects the degree of HIRI [7]. Mitochondrial DNA (mtDNA), believed to have bacterial origins, is released into the cellular matrix following ischemia-reperfusion, triggering the body's immune response, and exacerbating cellular damage [8]. Mitochondrial fusion and fission play a crucial role in regulating mitochondrial quality [9, 10]. The role of B lymphocytoma-2 (Bcl-2) family in regulating apoptosis and HIRI cannot be ignored either. A study showed that Bcl-2 overexpression can reduce the degree of HIRI [11]. An in-depth understanding of the mechanism of mitochondria in HIRI is hopeful to provide treatment options for reducing IRI caused by liver resection or liver transplantation, and improve the treatment effect and prognosis of patients. This article will review the mechanism of mitochondria in HIRI and the clinical research progress of drugs for the treatment of HIRI from the above five aspects.

## Mitochondrial autophagy and HIRI

The development of HIRI involves many kinds of cell death, with necrosis being the predominant death pathway; however, programmed cell death pathways such as apoptosis, ferroptosis, and cell scorching are also involved [12, 13]. Cell death associated with autophagy, a crucial biological mechanism, encapsulates damaged components through vesicle formation. It, then, fuses with lysosomes to form autolysosomes, facilitating the degradation of cytoplasm and organelles. This process supports cell metabolism and organelle renewal. Mitophagy eliminates excess or malfunctioning mitochondria, preserving the balance of mitochondrial quantity and mass, being critical for the functional integrity of the entire mitochondrial network and cell survival. The liver exhibits a high level of mitophagy. During the progress of ischemia-reperfusion, the regulation of mitochondrial autophagy and mitochondrial membrane stability are closely related. It has been shown that maintaining mitochondrial function by regulating mitochondrial autophagy can play a role in protecting hepatocytes and reducing HIRI [13, 14].

### PINK1-Parkin signaling pathway in mitophagy

**Figure 1.**
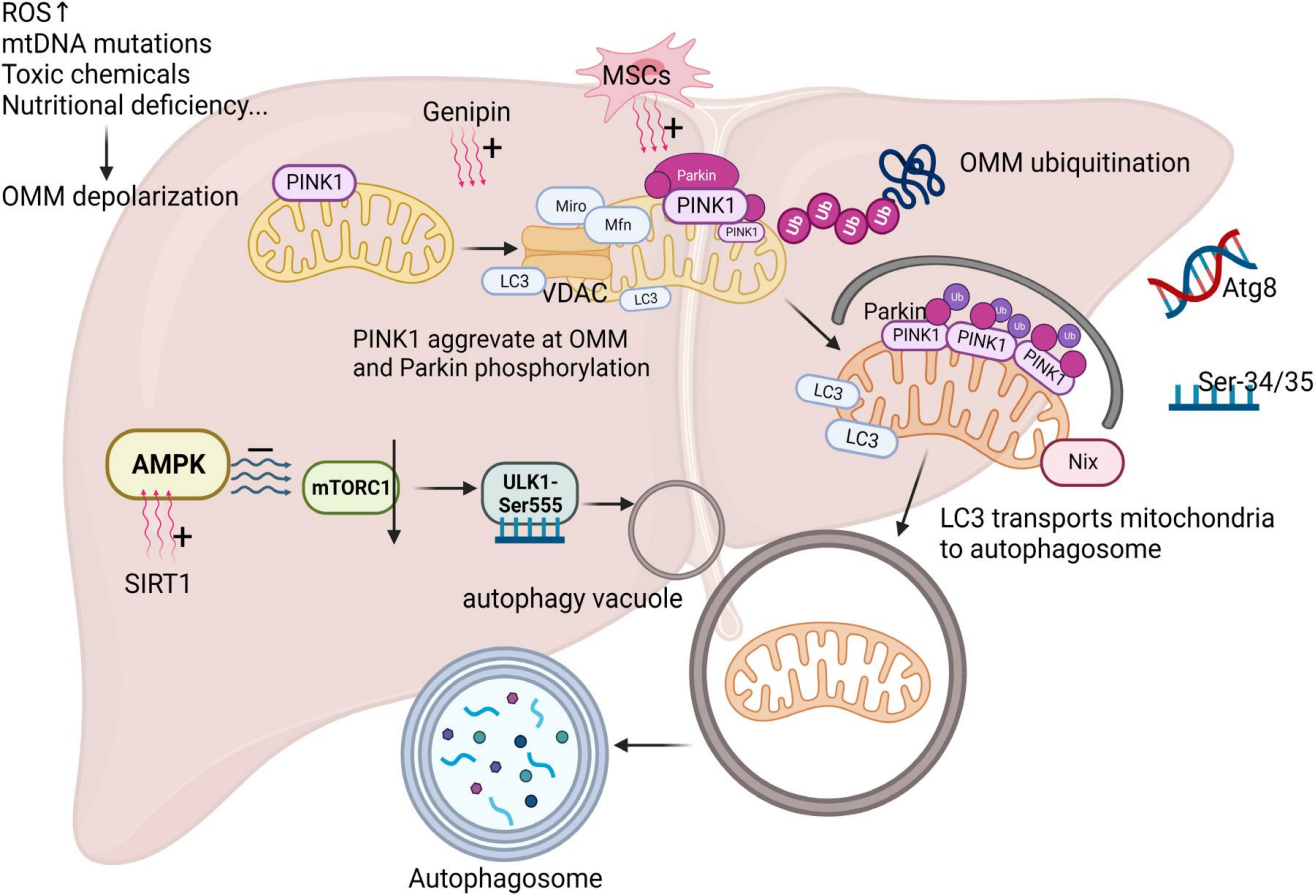
Mitochondrial autophagy. In the presence of elevated ROS, mtDNA mutations, chemical toxicant release, and nutrient deficiency, OMM depolarizes and PINK1 accumulates in the OMM, activating Parkin to ubiquitinate outer membrane proteins. LC3 transports the ubiquitinated mitochondria to the autophagosome and thereby mediates mitochondrial autophagy. Genipin and MSCs positively regulate the PINK1-Parkin pathway. AMPK activates the Ser555 site of ULK1 by inhibiting mTORC1, which contributes to autophagic vacuole formation, and SIRT1 facilitates this process. mtDNA = mitochondrial DNA, ROS = reactive oxygen species, OMM = outer mitochondrial membrane, IMM = inner mitochondrial membrane, MSCs = mesenchymal stem cells, AMPK = Adenosine 5′-monophosphate (AMP)-activated protein kinase, VDAC = voltage-dependent anion channels, LC3 = light chain 3, Nix = NIP3-like protein X, ULK1 = Unc-51-like autophagy initiation kinase 1.

Mitophagy encompasses various signaling pathways, e.g. PINK1-Parkin, BNIP3/NIX, and FUNDC1, with the PINK1-Parkin pathway being a major concern of current research. Phosphatase and tensin homolog-induced putative kinase 1 (PINK1) is a protein kinase encoded by the *PARK6* gene. PINK1 exhibits broad expression in human cells, particularly in high energy-consuming organs. Parkin, encoded by the *PRKN* gene, is exceptionally large at 1.38 million base pairs, making it one of the largest genes in humans. Human Parkin is a 465-amino acid protein serving as an E3 ubiquitin ligase. It mediates the attachment of ubiquitin molecules to target proteins, marking them for protease-mediated degradation. Ubiquitin-mediated mitochondrial autophagy relies on the interaction between PINK1 and Parkin. PINK1 primarily resides in the outer mitochondrial membrane and is continually recruited to the inner mitochondrial membrane under normal conditions. It is then rapidly degraded by the ubiquitin-proteasome system [15]. In case of mitochondrial damage, the mitochondrial membrane potential decreases, hindering the translocation of PINK1 into the inner mitochondrial membrane, resulting in its accumulation within the outer mitochondrial membrane [16]. The accumulated PINK1 in the outer mitochondrial membrane undergoes autophosphorylation, allowing for the replenishment of Parkin in the damaged mitochondrial outer membrane and activating the ubiquitin ligase function of Parkin. Parkin can ubiquitinate mitochondrial outer membrane proteins, and substrates of Parkin such as mitofusin (Mfn), mitochondrial Rho GTPase (Miro), and voltage-dependent anion channels (VDAC) are polyubiquitinated. This is because its E3 ligase activity is recognized by an adaptor protein that is anchored to microtubule-associated protein 1A/1B light chain 3 (LC3) in the phagosome. LC3 facilitates mitophagy by transporting ubiquitinated mitochondria to autophagosomes. As a key protein for autophagosome generation and a marker protein on the autophagosome membrane, it is of significance for responding to the autophagic process. The receptor-mediated clearance of mitochondrial phagocytosis involves the Bcl-2 family of proteins, and these mitochondrial phagocytosis receptors are localized to the outer mitochondrial membrane and interact with LC3 to initiate mitochondrial autophagy and degradation [17, 18] (Figure 1).

Under normal physiological conditions, cells eliminate damaged or dysfunctional mitochondria through autophagy. However, during ischemia-reperfusion events, mitochondrial autophagy exhibits a dual nature. During ischemia, rational mitochondrial autophagy effectively repairs damaged mitochondria, suppressing the release of substantial amounts of reactive oxygen species (ROS), cytochrome C (cyt-C), and other pro-apoptotic substances [19]. Following the initial hypoxic state, during reperfusion, a specific amount of ROS directly damages mitochondria, leading to oxidative stress and the activation of PINK1/Parkin pathway-mediated autophagy, resulting in severe cellular damage [20].

Although nutrient depletion during prolonged ischemia is the trigger for autophagy, the energy deficiency that accompanies autophagy is the main factor in its impairment. The absence of adenosine triphosphate (ATP) may prevent the formation of autophagic vesicles and inhibit mitochondrial autophagy [21]. Moreover, the presence of PINK1 and Parkin inhibits ATP depletion, enhancing the effect of supplementation-induced apoptosis. In other words, mitochondrial stability can be maintained by regulating related factors such as PINK1 and Parkin, thus, mitigating the damage suffered by the liver during the ischemia-reperfusion phase due to mitochondrial dysfunction [22].

### AMPK pathway in mitophagy

The PINK1/Parkin pathway is widely studied, whereas the AMPK pathway is an alternative regulator of mitophagy. AMPK is a vital kinase for regulating energy homeostasis and plays a central role in biological energy metabolism. It is present in multiple signaling pathways and can be activated by various physiological stimuli. AMPK activation suppresses mTOR complex 1 (mTORC1) by targeting the Ser555 site of the Unc-51-like autophagy-activated kinase 1 (ULK1) complex, initiating autophagic vacuole formation, signifying the commencement of autophagy [23, 24]. Simultaneously, AMPK regulates the generation of new mitochondria via PGC-1α transcription [25]. This dual regulatory process by AMPK enables newly formed mitochondria to replace dysfunctional ones, achieving a “mitochondrial purification effect” critical in numerous physiological and pathological processes. The activation of the AMPK/ULK1 pathway by isoflurane significantly attenuates HIRI, while the inhibition of AMPK phosphorylation exacerbates liver injury and oxidative stress in ischemia-reperfusion injured mice [26, 27]. In previous studies, Genipin was shown to upregulate hepatocyte mitochondrial autophagy in a PINK1-dependent manner during ischemia-reperfusion injury; inhibition of sirtuin 1 (SIRT1) was observed to decrease AMPK phosphorylation and diminish the effect of Genipin on HIRI [28, 29]. SIRT1 is an NAD^+^-dependent type III protein deacetylase known for its role in regulating hepatic lipid metabolism and inflammation, promoting autophagy, and restoring mitochondrial quantity and membrane potential [30]. SIRT1 is thought to induce mitochondrial autophagy by directly deacetylating the Mfn2 located in the outer mitochondrial membrane.

### Other factors in mitophagy

Mitochondrial autophagy process involves various pathways. For example, Zhou *et al*. [31] demonstrated that the knockdown of the CCAAT/enhancer binding protein homolog (CHOP) reduces hepatocyte death during HIRI. CHOP deletion also enhances mitochondrial autophagy by upregulating dynamin-related protein 1 (Drp1) and BECN1 expression in mice after ischemia-reperfusion. In addition, NIP3-like protein X (Nix) is a significant mediator of mitochondrial autophagy. It binds to LC3 as an autophagosome receptor through phosphorylation at Ser34/35, or it recruits the autophagy-related gene (Atg8) protein family to damaged mitochondria, inducing autophagy [32]. Moreover, Krüppel-like factor 10 (KLF10) serves as a transcriptional regulator and a key component of the TGF-β pathway. KLF10 is intricately linked with oxidative stress, autophagy, and IRI, and also plays an important role in a variety of liver diseases [33]. KLF10 diminishes apoptosis by directly activating mitochondrial autophagy through binding to the promoter of Parkin, thus, alleviating HIRI. Under oxidative stress, HIF-1α initiates KLF10 transcription by binding to the promoter of KLF10, facilitating mitochondrial autophagy through the HIF-1α/KLF10/Parkin axis to reduce apoptosis and HIRI. Knockdown of KLF10 reduced mitochondrial autophagy in mouse HIRI model, exacerbating liver function impairment, highlighting the clinical translational value of KLF10. Whereas, high KLF10 expression can expedite liver function recovery following liver transplantation, which has some clinical application prospects. Zheng and colleagues [34] demonstrated that as liver injury improved, mesenchymal stem cells (MSCs) demonstrated the ability to regulate mitochondrial mass in a HIRI mouse model and L02 hepatocyte hypoxia/reoxygenation model. MSCs can reduce the overproduction of mitochondrial ROS, restore the antioxidant system, minimize the buildup of mitochondrial debris, boost ATP production, and enhance mitophagy. Furthermore, they observed that MSCs reversed the decrease in Parkin and PINK1 expression and the inactivation of the AMPK pathway in the HIRI model. In other words, MSCs provide hepatoprotective effects during HIRI by enhancing PINK1-dependent mitochondrial autophagy.

### Application of clinical drugs to mitophagy

Some of the drugs that affect mitochondrial autophagy and their mechanisms are listed in Table 1. It is essential to noted that while these drugs have received some research support, the mechanisms and effects of drugs may be different in different individuals or under different experimental conditions. Therefore, further studies are required to enhance our understanding of these mechanisms.

**Table 1.** Some drugs that affect mitophagy and their effects

Drugs	Regulated molecules/ions/genes	Methods	Effects on mitochondrial autophagy	References
Rapamycin	mTOR, Bcl-2, Fas-mRNA, cyt-c, ULK1	↑Bcl-2 mRNA level ↓Fas-mRNA level; ↓cyt-C; reduces mtDNA damage; ↓mTORC1 activity, ↑ULK1 activity and promotes autophagic vesicle formation	Promote	[28, 35–38]
Metformin	AMPK	Obstructs MRCC, activates AMPK pathway	Promote	[39–41]
CCCP	AMPK, PINK1/Parkin	Disrupts the mitochondrial electrochemical gradient and activates the PINK1/Parkin pathway	Promote	[42]
Mdivi-1	Drp-1 (Ser616), caspase-3, cyt-C	↓mitochondrial Drp-1 protein level; ↓mtDNA fragmentation; ↓caspase-3 protein level; ↓cytochrome C protein and mRNA levels	Suppress	[43–47]
Valinomycin	AMPK, K^+^	Interferes with K^+^ balance and promotes mitochondrial swelling	Promote	[48]
Chloroquine	H^+^	inhibits lysosomal acidification and interferes with the degradation of mitochondria by autophagosomes.	Suppress	[49, 50]
Oxaliplatin	mtDNA, ion pump	Binds to mtDNA and leads to mtDNA damage; produces ROS; interferes with membrane potential	Promote	[51, 52]

MRCC = mitochondrial respiratory chain complex, CCCP = carbonyl cyanide-m-chlorophenylhydrazone, mtDNA = mitochondrial DNA, ROS = reactive oxygen species, AMPK = Adenosine 5’-monophosphate (AMP)-activated protein kinase, Drp1 = dynamin-related protein 1, mTOR = mechanistic target of rapamycin, ULK1 = Unc-51-like autophagy initiation kinase 1.

## Opening of the mitochondrial MPTP and HIRI

The autophagy described above is a component of apoptosis. Will the mitochondrial membrane potential change during apoptosis? The answer is yes. The mitochondrial membrane contains numerous channels regulating material transport, collectively referred to as the voltage-dependent MPTP. It is a group of nonspecific protein complexes present between the inner and outer mitochondrial membranes. The outer mitochondrial membrane contains various pore proteins, each featuring a channel permitting molecules weighing <5 kDa to pass. This channel facilitates the transport of large mitochondrial membrane transfer proteins [53]. Upon stimulation by apoptosis signals, the mitochondrial MPTP channel opens, resulting in heightened mitochondrial membrane permeability and a decline in the mitochondrial trans-membrane potential. MPTP is deactivated during ischemia and reactivated during reperfusion, earning it the name “apoptotic switch” due to its dynamic behavior [54–56]. Mechanisms associated with HIRI, including oxidative stress, calcium overload, and specific signal pathways linked to mitophagy, are connected to the opening of the mitochondrial MPTP. We will provide an overview of each of these below.

### Oxidative stress and MPTP

In hepatic ischemia-reperfusion, substantial ROS production occurs, leading to changes in the mitochondrial trans-membrane potential and the opening of the MPTP [57]. ROS can interact with polyunsaturated fatty acids in biological membranes, resulting in the generation of highly reactive molecules. This process leads to lipid peroxidation and the release of harmful aldehydes such as 4-hydroxynonenal and malondialdehyde. These compounds can significantly disrupt normal apoptotic signaling pathways and cause destruction [58–60]. Studies on ferroptosis in recent years have shown that iron has a catalytic effect on ROS formation and can stimulate MPTP opening to induce apoptosis. During ischemia, Fe^2+^ is released from intracellular lysosomes and transported into mitochondria through the unidirectional transporter protein complex. The results in iron overload and upon reperfusion with hydrogen peroxide, a significant amount of hydroxyl radicals is generated. These radicals damage mtDNA, proteins, and cell membranes, ultimately causing MPTP opening and apoptosis. In addition, Nakazato *et al*. [61] indicated that protein kinase C has a strong indirect elevating effect on nuclear transcription factor kappa B (NF-κB) activation and ROS production, thus, promoting MPTP opening.

### Calcium overload and MPTP

Calcium (Ca^2+^), a crucial cellular signaling molecule, serves as a ubiquitous second messenger and a cofactor for various enzymes. It plays a significant role in maintaining intracellular homeostasis. Mitochondria and endoplasmic reticulum are the primary sites for the rapid and localized release of calcium during specific signaling processes. The mitochondrial trans-membrane potential reflects the state of calcium ion accumulation [62]. Excessive calcium accumulation will disrupt cellular homeostasis and initiate a cascade of cellular damage. Ca^2+^ transportation in mitochondria is mediated by three structures: the Ca^2+^ mitochondrial calcium uniporter (MCU), the Na^+^/Ca^2+^ exchanger (NCLX), and the Ca^2+^/H^+^ reverse transporter protein (Letm1) [63]. When ischemia and hypoxia occur, the functions of all three transporters are compromised, leading to the calcium overload. Ischemia and hypoxia increase cell membrane permeability, causing intracellular Ca^2+^ accumulation. Upon reperfusion with oxygen, the MPTP opens and releases large amounts of stored calcium ions, triggering apoptosis. Several studies have explored the connection between Ca^2+^ and MPTP. Some researchers have proposed that hypoxia interferes with oxidative phosphorylation to impede ATP production, leading to Ca^2+^ overload. This, in turn, exacerbates lipid peroxidation and further attenuates the oxidative phosphorylation process, establishing a positive feedback loop that depletes ATP [64]. Concurrently, excessive Ca^2+^ overload increases the extent of mitochondrial Na^+^/Ca^2+^ exchange and inactivation states, resulting in a higher Ca^2+^ concentration that is adequate to induce MPTP opening, consequently compromising mitochondrial membrane integrity [65, 66]. A large number of mitochondria can be found near the site of calcium release, allowing for precise control of local ion concentration and ensuring effective regulation of local calcium effects [67].

### PINK1-Parkin signaling pathway and MPTP

It has been suggested that alterations in mitochondrial transmembrane potential can serve as a precursor to autophagy PINK1 can accumulate on the outer mitochondrial membrane, initiating the selective translocation of Parkin, which contributes to autophagy and effectively eliminates scathing mitochondria [68]. Zhou and Zhang [27] found that melatonin blocked mitochondrial autophagy-mediated cell death by inhibiting the opening of MPTP and suppressing the activation of PINK1/Parkin. Rodriguez-Enriquez *et al*. [69] found that starvation-induced mitochondrial autophagy could be inhibited by the MPTP opening inhibitor ciclosporin A (CsA) in hepatocytes cultured *In vitro*. Recent studies have revealed that the expression level of MPTP can be significantly reduced by inhibiting the expression of the mouse *Prif* gene (the gene encoding procyclin D [CypD]) [70].

The mitochondrial permeability transition cycle involves MPTP switching, mitochondrial membrane polarization, and the resting phase. The interplay between these cycles significantly impacts the pathological response of hepatocytes in ischemia-reperfusion. It was shown that ginsenoside Rg1 modulates the opening and closing of MPTP by inhibiting CypD protein expression. This inhibition curtails the mitochondrial apoptotic pathway, ultimately mitigating HIRI. Furthermore, Rg1 inhibits excessive mitochondrial autophagy during the reperfusion phase by partially suppressing CypD protein expression levels. This stabilization of the mitochondrial trans-membrane potential, further limits the PINK1-Parkin signaling pathway-mediated mitophagy [71]. In summary, proper mitochondrial permeability transition cycle regulation can help exert the defense role of mitophagy, maintain mitochondrial stability, and alleviate hepatocyte death during ischemia-reperfusion injury.

## Damage and release of mtDNA in HIRI

**Figure 2.**
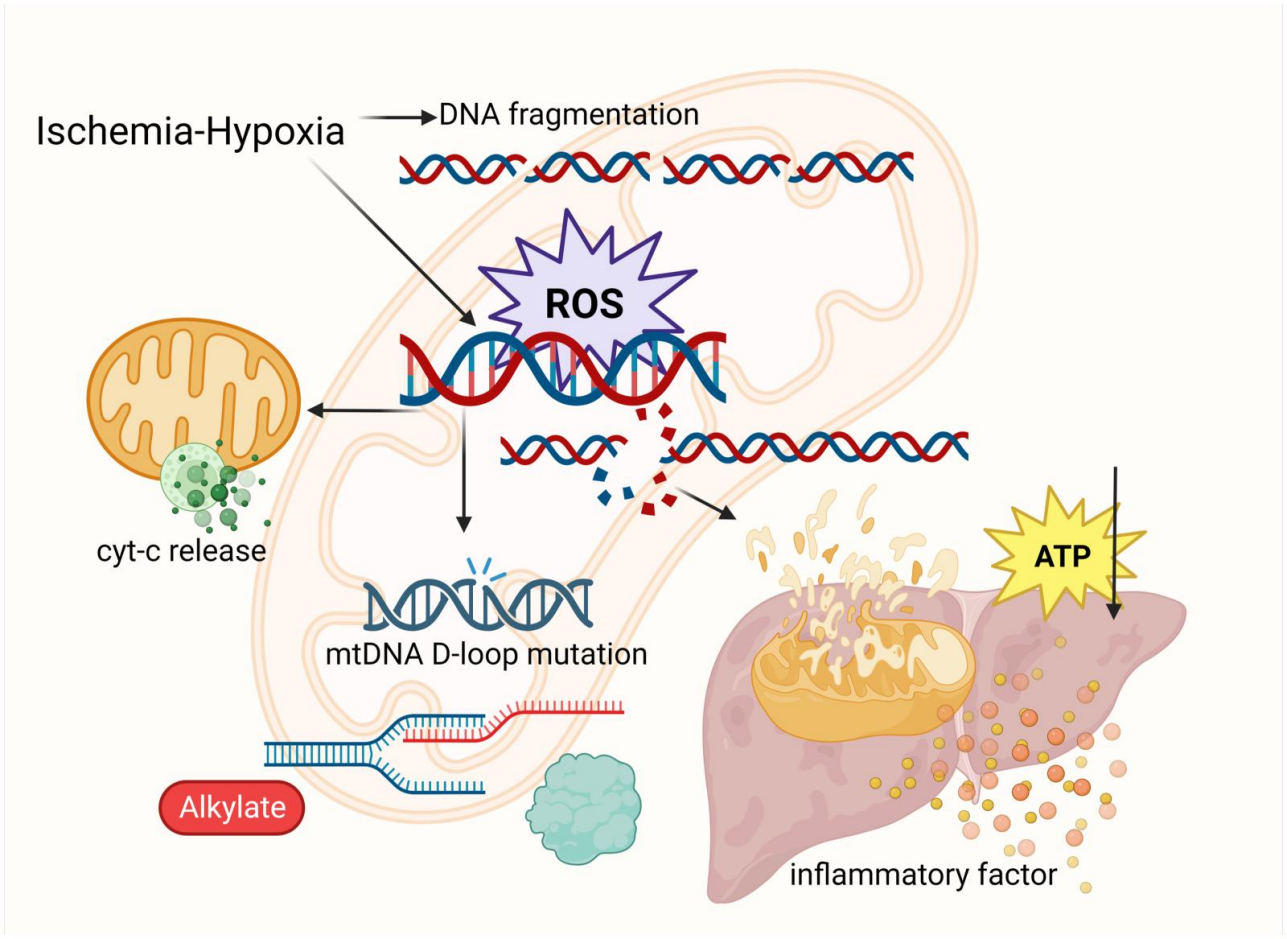
Factors affecting mtDNA release and the impact on HIRI. During ischemia and hypoxia, mitochondrial DNA is fragmented, exposing mtDNA to ROS for a long time and DNA break, which on the one hand allows cyt-C to be released from the mitochondria and on the other hand mutations occur in the D-Loop region, affecting gene expression and ATP production. In addition, the mtDNA undergoes rupture, and cytokines are released, allowing for the intensification of the inflammatory response. mtDNA = mitochondrial DNA, ROS = reactive oxygen species, cyt-C = cytochrome C, HIRI = hepatic ischemia-reperfusion injury.

The MPTP regulates the passage of certain ions in and out of mitochondria, raising the question of how mtDNA, originally located within mitochondria, can be damaged and released. This issue remains a subject of ongoing investigation. Since mtDNA may have originated from bacteria, it is perceived as “foreign” material within the cell, distinct from the cell's own DNA. A study has noted that mtDNA exhibits lower levels of methylation when compared with nuclear DNA [72]. mtDNA is situated in the mitochondrial matrix, making it particularly vulnerable to oxidation by ROS in the electron transport chain. This oxidative stress can result in mtDNA mutations. The occurrence of HIRI is inextricably linked to the destruction of mtDNA. Studies have reported that both mitochondrial number and mtDNA content decrease during HIRI [29, 73]. Genetic mutations or absence of mtDNA disrupt the respiratory chain, impacting mitochondrial energy metabolism and ultimately leading to severe liver injury. HIRI causes oxidative stress, resulting in a decrease in mitochondrial potential and a decrease in the activity of the internal mitochondrial enzyme system, which in turn induces the release of mtDNA. However, the excessive release of mtDNA and the ensuing inflammatory response usually form a vicious circle, aggravating the degree of ischemia-reperfusion injury. DNA fragmentation occurs when intracellular levels of ROS are elevated. However, mtDNA lacks protection from histones and lacks an effective DNA repair mechanism. Therefore, they are continuously exposed to the ROS environment, which enhances their destructive capacity and consequently reduces ATP production [74]. The damage to mtDNA results in the release of cyt-C from mitochondria into the cytoplasm, subsequently inducing apoptosis in hepatocytes. As the duration of ischemia lengthens, the expression of cyt-C increases and intensifying the degree of apoptosis. In a study by Guo *et al.* [75], there were 62 mutant sites in the mtDNA D-loop region in HIRI. This highlights the high sensitivity of mtDNA to ischemia and hypoxia, with mutation rates increasing with prolonged ischemia. Furthermore, mutations in the mtDNA D-loop region will be bound to affect the transcription and replication of mtDNA, worsening hepatic injury [76]. During HIRI, the cessation of ATP synthesis during ischemia can lead to mitochondrial oxidative stress and mtDNA alkylation damage. These damages will be further aggravated with reperfusion (Figure 2). Alkylation damage primarily results from the interaction of oxides produced during mitochondrial oxygen consumption with deoxyribose and its bases in mtDNA. This type of DNA damage is common. Alkylation damage can impair mtDNA replication and transcription, disrupting the regular epistatic regulation of ATP synthesis-related proteins synthesized by mitochondrial genes. This disruption inhibits ATP synthesis and worsens hepatocyte injury. In addition, mtDNA damage can also give rise to spontaneous mitochondrial rupture, which in turn contributes to the massive release of cytokines and an exacerbation of inflammatory responses, thus, aggravating HIRI. DNA polymerase γ is a key element in mtDNA reconstruction as the level of its mRNA expression serves as an indicator of the extent of mtDNA damage.

In response to the role of mtDNA in HIRI, we may consider setting about developing some targeted therapeutic drugs in this direction. Rapamycin has the capacity to reduce mtDNA base damage and low the DNA mutation rate. It also effectively suppresses the overexpression of mtDNA polymerase γ mRNA in HIRI, ensuring normal mtDNA replication, translation, and transcription. Furthermore, it enhances mtDNA repair and mitigates hepatocyte apoptosis. These combined effects reduce the extent of hepatocyte injury during ischemia-reperfusion [28, 35]. Aberrant mtDNA release as a driver or participant in disease is implicated in a wide variety of pathological processes. Gaining insights into how mtDNA specifically excludes mitochondria, for example, whether it can be associated with MPTP, will provide us with new therapeutic avenues.

## Mitochondrial fission

**Figure 3.**
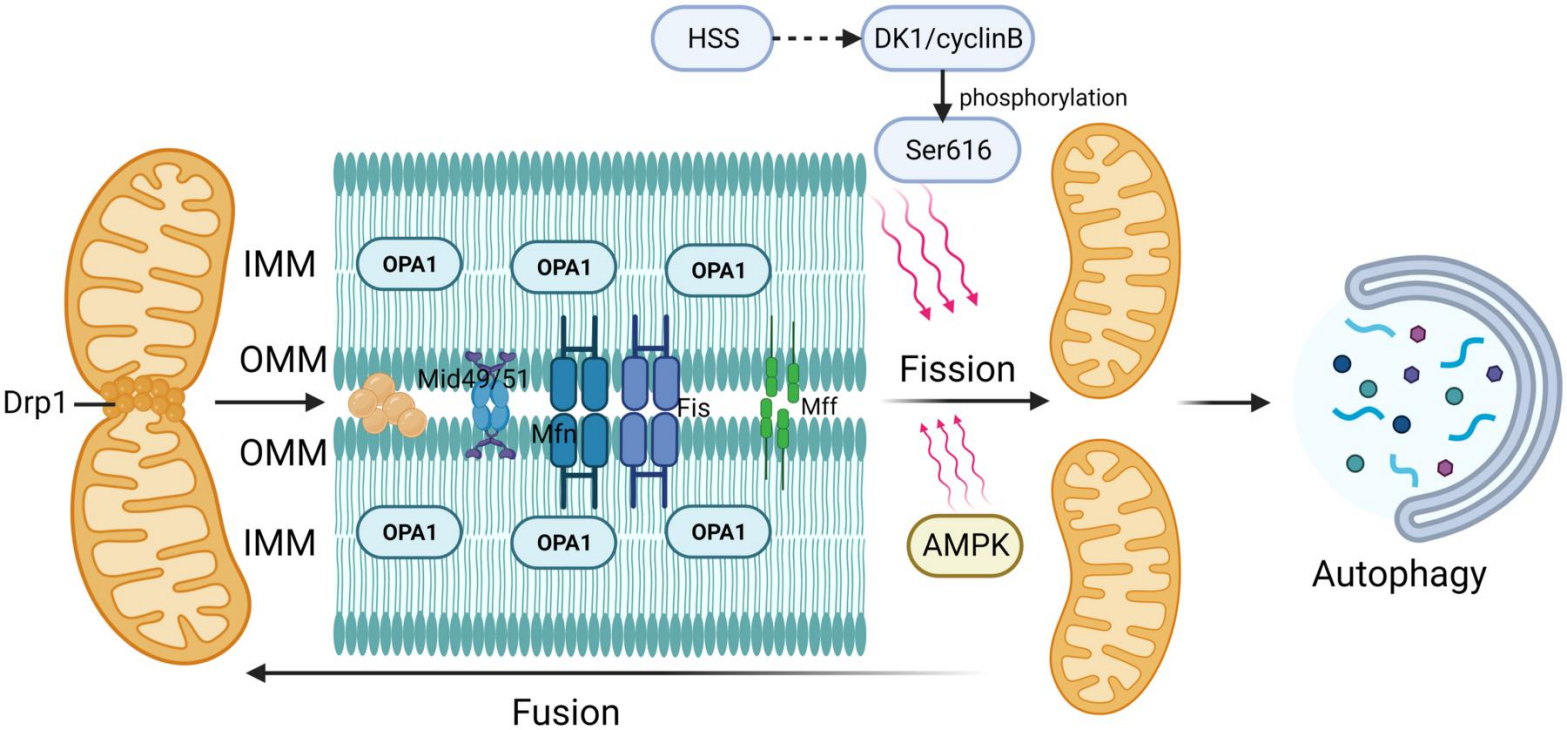
Mitochondrial fission and fusion. Drp1, Mff, Mid49, Mid51, and Fis1 in the OMM interact with each other to cause mitochondrial fission. OPA1 is located in the IMM and mediates fusion. HSS can inhibit the expression of DK1/cyclin B and suppress the phosphorylation of Drp1 at the Ser616 site to inhibit mitochondrial fission. Drp1 = dynamin-related protein 1, Mff = mitochondrial fission factor, Mfn = mitochondrial fusion protein, Fis = mitochondrial fission protein, Mid49/51 = mitochondrial dynamin49/51, OPA1 = optic atrophy 1, OMM = outer mitochondrial membrane, IMM = inner mitochondrial membrane, HSS = hepatic stimulation substance, AMPK = Adenosine 5′-monophosphate (AMP)-activated protein kinase.

The human body is a dynamic and balanced system. It possesses a regulatory capacity in response to external stimuli, however, surpassing its limits can result in pathological injury. The previously mentioned mitochondrial damage occurs due to the disruption of the initially normal physiological processes after ischemia-reperfusion. Mitochondria keep their dynamic renewal and homeostasis by fission and fusion, and either redundant mitochondrial fission or reduced mitochondrial fusion will cause variations of mitochondrial dynamics that affect mitochondrial function and have a negative impact on HIRI [77–79]. Following mitochondrial division, damaged organelle fragments fuse with each other to form a reticular system to renew damaged mtDNA. Excessive fission initiates the mitochondrial apoptotic pathway, leading to a lack of mitochondrial stability and enzymatic activity in hepatocytes. This imbalance in the energy metabolism of hepatocytes exacerbates ischemia-reperfusion injury [65]. It has been indicated that there is a positive feedback interrelationship between cyt-C release and mitochondrial fission, and the two promote each other [80]. According to the study, mitochondrial dynamics perhaps have to do with the regulation of Drp1, mitochondrial fission protein 1 (Fis1), Mfn1/2, and mitochondrial fission factor (Mff) [81]. Drp1 is a critical protein for mitochondrial division, and it is primarily located in the cytoplasm. Following translational modification in the cytoplasm, Drp1 will be transferred to the outer mitochondrial membrane, where it interacts with Mff, mitochondrial dynamic proteins (Mid49, Mid51), and Mfn1 to mediate the division of the outer membrane [80, 82, 83]. Interruption this pathway shields hepatocytes from IRI-induced apoptosis [84]. Mitochondrial fusion is thought to be a defense response in which two mitochondria merge into one to enhance their resistance properties. Mitochondrial fusion consists of outer membrane fusion mediated by Mfn1 and Mfn2 in the outer mitochondrial membrane and inner membrane fusion facilitated by the inner membrane protein optic atrophy 1 (OPA1) in the inner mitochondrial membrane (Figure 3). Likewise, the Ser616 phosphorylation of Drp1 contributes to mitochondrial division and phosphorylation at the Ser637 site leads to apoptosis. The compound DK1/cyclin B has positive effects on Drp1 phosphorylation at the Ser616 site. In contrast, hepatic stimulation substance significantly inhibits the expression of cyclin-dependent kinase 1 (CDK1) and Bax, which reduces Drp1 phosphorylation at the Ser616 site and releases cyt-C to resist HIRI [84]. Phosphorylation of the Drp1 Ser637 site by protein kinase A extends the mitochondrial length and inhibits apoptosis. Whereas, dephosphorylation of the Drp1 Ser637 site by calcineurin promotes the fragmentation of mitochondria [85].

Mitochondrial fission can also lead to the accumulation of intracellular free radicals and the activation of autoimmune responses, causing inflammatory responses and hepatocyte apoptosis and contributing to the development and progression of hepatic IRI. Hence, controlling and regulating mitochondrial quantity and homeostasis in hepatocytes with the use of mitochondrial protective agents or anti-mitochondrial fission drugs could emerge as a new way to treat HIRI. For example, it has been reported that augmenter of liver regeneration can inhibit the phosphorylation of Drp1 to exert a protective effect, while ubiquitin-like modification (SUMOylation) can promote Drp1 activation to accelerate mitochondrial division [86]. It has been confirmed that the hormone irisin could protect mice from HIRI by inhibiting Drp1 and Fis1, increasing mitochondrial biogenesis, and suppressing apoptosis [87].

## Regulation of HIRI by Bcl-2 family proteins

**Figure 4.**
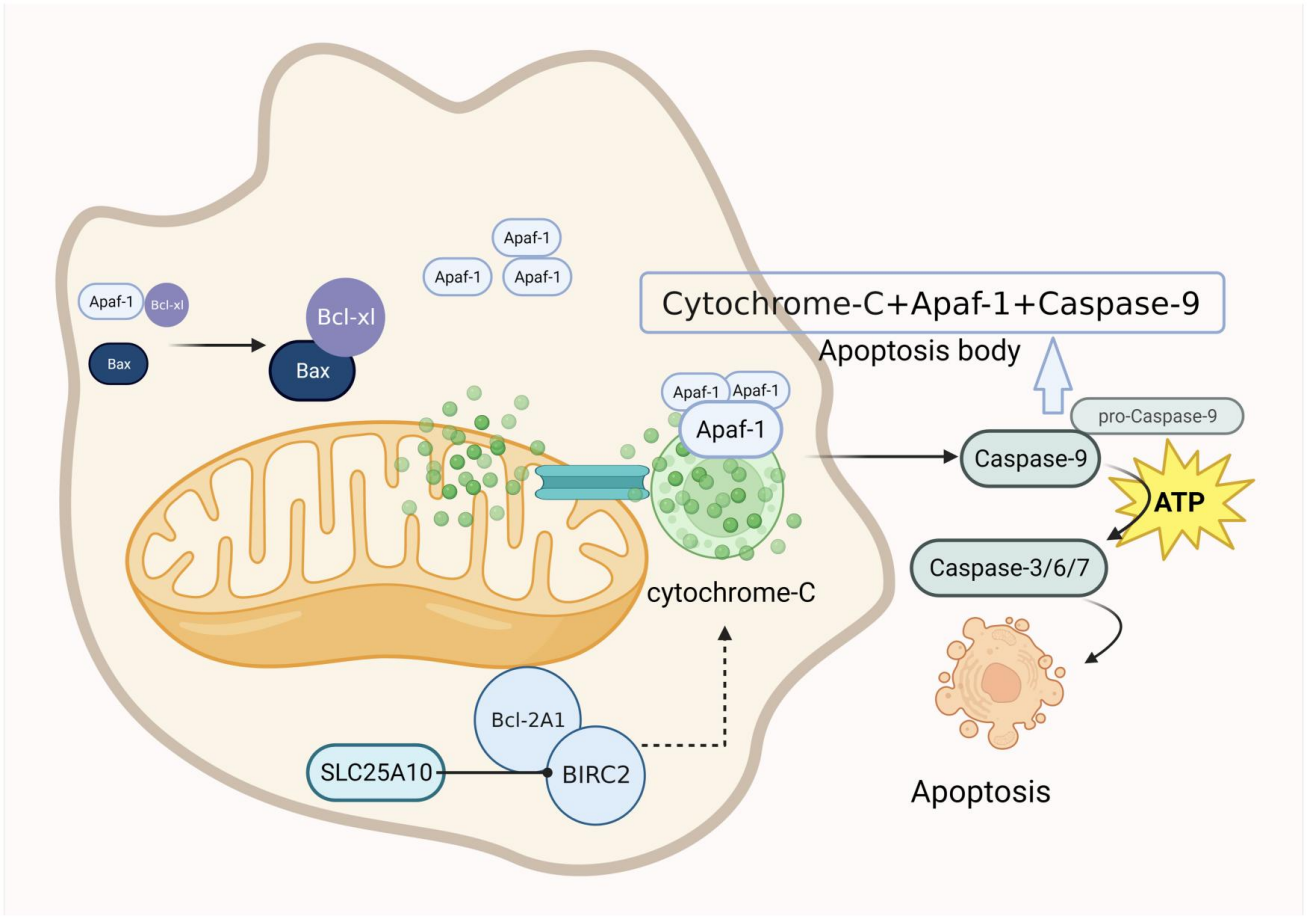
Mitochondrial apoptosis pathway mediated by Bax/Bcl-xl and caspase family. Bax binds to Bcl-xl to free Apaf-1 in the cytoplasm and mitochondria release cytochrome C into the cytoplasm, making cytochrome C interact with Apaf1 to activate caspase 9. In addition, cytochrome C, Apaf-1, and caspase 9 together form the apoptosis body. With the help of ATP, they promote apoptosis to occur. SLC25A10 can reduce the release of cytochrome C from mitochondria, and it inhibits the activation of downstream signaling pathways by regulating Bcl-2A1 and BIRC2. Apaf-1 = apoptosis protease-activating factor 1, SLC25A10 = solute carrier family 25 member 10, BIRC2 = baculoviral IAP repeat containing 2.

Exploring these mitochondrial destruction mechanisms reveals that mitochondrial matrix swelling, loss of endosomal cristae, and the disintegration of the endosomal membrane caused by damage result in changes in membrane permeability. This leads to the release of apoptosis-related proteins, including cyt-C (a trigger of apoptosis), into the cytoplasm [88, 89]. Consequently, this triggers the cascade reaction of aspartate-specific cysteine proteases (Caspases), initiating intrinsic apoptosis [90, 91]. This represents a classical apoptotic pathway, functioning as both the upstream pathway of mitochondrial apoptosis and a central element in the Caspase pathway [92]. The Caspase family operates in the later stages of apoptosis regulation, with their heightened expression and activation playing a crucial role in governing mitochondrial apoptotic signaling pathways [93, 94]. The Bcl-2 family is closely associated with the regulation of apoptosis and participates in the pathogenesis of different kinds of diseases, including HIRI. It was found that Bcl-2 can effectively inhibit upstream Caspase protease activity, thus diminishing apoptosis [95]. Bcl-2 and Bcl-xl are significant proteins that inhibit and reduce the level of apoptosis and subsequently reducing the extent of cell injury. In contrast, Bax and Bak are beneficial to apoptosis. Bax, as an important component of mitochondrial membrane channels, can activate Caspase-9 on mitochondrial membranes, enhancing Caspase-3 activity and expediting the onset of apoptosis. Under normal conditions, Bcl-xl binds to apoptosis protease-activating factor 1 (Apaf-1) so that Apaf-1 is not involved in the activation of Pro-caspase-9 [96]. Bax interacts with Bcl-xl when cells are stimulated by death signals, removing the inhibitory effect of Bcl-xl on Apaf-1. This leads to the release of cyt-C from mitochondria, its binding to Apaf-1, and the replacement of ADP with ATP/dATP, activating Apaf-1 free in the cytoplasm. In turn, Pro-caspase-9 is activated by its hydrolysis to become caspase-9. The apoptosome is made up of cyt-C, Apaf-1, and Caspase-9. Caspase-9 gets activated by its self-splicing and then it triggers the Caspase cascade reaction, which continues to activate members of Caspase-3, 6, and 7 with the help of dATP and ATP so that apoptosis is allowed to proceed [97] (Figure 4).

In the mechanism of mitochondria-mediated apoptosis, Bcl-2 family proteins can not only effectively alter the permeability of mitochondria and form pores to expel substances from mitochondria but also activate the activity of mitochondrial MPTP, effectively boosting the release of apoptosis-associated proteins. Yang *et al*. [98] found that pre-treatment with octreotide reduced the activity of mitochondrial metabolic enzymes and alleviated HIRI, which includes NADH_2_ dehydrogenase and prolyl phosphate isomerase. Their previous study also indicated that the protective mechanism of octreotide is probably related to the decrease in endotoxin levels, the downregulation of inflammatory cytokines (TNF-α and IL-1β), the inhibition of hepatocyte apoptosis, and the Bcl-2/Bax-mediated mitochondrial apoptotic pathway [99]. Lin *et al*. [100] reported that SLC25A10 participates in the mitochondrial apoptotic signaling pathway by regulating Bcl-2A1 and baculoviral IAP repeat containing 2 (BIRC2). This regulation reduces the release of cyt-C from mitochondria, and inhibits the activation of downstream signaling pathways. Inhibiting Bcl-2, an inhibitor of apoptosis, reduces ATP production by suppressing the tricarboxylic acid cycle and mitochondrial respiration [101]. It has been shown that Rapamycin has a prominent role in reconstruction by inhibiting the activation of several cytokines, suppressing the expression of the pro-apoptotic gene Fas-mRNA, and promoting the expression of the apoptosis-inhibiting gene Bcl-2 mRNA to protect the outer mitochondrial membrane from damage. Rapamycin inhibits the release of cyt-C, produces intracellular antioxidant effects, inhibits apoptosis, maintains the normal structural function of mitochondria, reduces respiratory chain dysfunction brought about by hypoxia in ischemia-reperfusion, and enhances cellular adaptation to hypoxic environments [28, 35].

## Summary and future perspectives

The current study has improved our comprehension of the role of mitochondria in HIRI. Mitochondria are intricately involved in numerous metabolic and signaling pathways within the human body. This article only briefly discusses the regulation of mitophagy, MPTP, mtDNA damage, and Bcl-2 family. The regulatory genes, proteins, and signaling molecules involved in each mechanism are interconnected and interact extensively. Besides the molecules discussed in this article, numerous other regulatory factors, such as PGC-1α, play roles in mitochondrial biogenesis and cellular adaptation [102]. Currently, drugs targeting PGC-1α, like honokiol, have demonstrated the potential to mitigate ischemia-reperfusion by modulating the AMPK/PCG-1α/SIRT3 pathway [103]. Other natural substances have also been shown to have protective effects on mitochondrial function. Perhaps we can not only study small molecule drugs and protein drugs but also look for natural macromolecular compounds as much as possible [104, 105].

For patients with liver failure and liver injury, *In vitro* mitochondrial transplantation may be a good option alongside drug therapy and organ transplantation [106–108]. In addition, instrumental perfusion can detect mitochondrial function and related markers, and the use of mechanical perfusion to mitigate mitochondrial damage can improve graft function to some extent [109]. Utilizing oxygenated solution flushing during liver transplantation to buffer against hypoxia and mitigate mitochondrial damage may be an effective intervention to mitigate IRI. At a time when the organ donor pool is in such short supply, we need to make more breakthroughs in the mechanism of ischemia-reperfusion occurrence in addition to expanding the standard donor. And the study of mitochondria is undoubtedly an important avenue, especially the study of key molecules in its biogenesis, adaptation, and mass regulation and kinetic modulation. In the future, a large number of studies are still needed to substantiate hypotheses and establish a therapeutic foundation for mitigating HIRI.

## Funding

This study was supported by the National Natural Science Foundation of China [grant number 81870067, 82170664].

## Conflicts of Interest

No conflict of interest, financial, or otherwise, are declared by all the authors.
